# Light, Reflection, Illumination

**DOI:** 10.3201/eid2106.AC2106

**Published:** 2015-06

**Authors:** Byron Breedlove

**Affiliations:** Centers for Disease Control and Prevention, Atlanta, Georgia, USA

**Keywords:** art science connection, emerging infectious diseases, cholera, antimicrobial resistance, August Köhler, Köhler illumination, One-Health, art and medicine, Joaquin Sorolla y Bastida, The Wounded Foot, Light, Reflection, Illumination, about the cover

**Figure:**
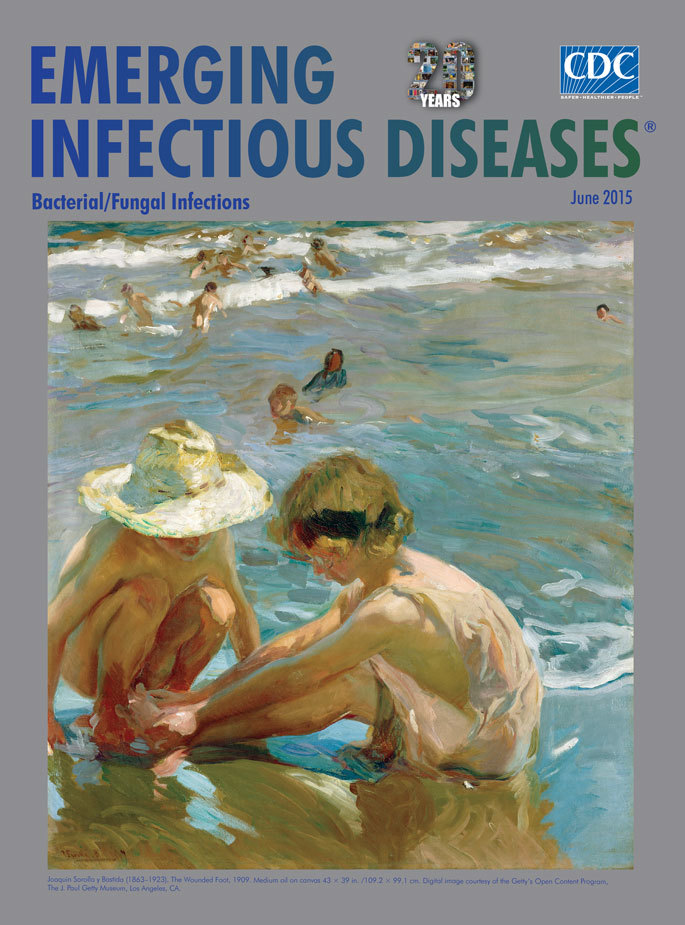
**Joaquin Sorolla y Bastida (1863–1923). The Wounded Foot, 1909. Medium oil on canvas 43 × 39 in. /109.2 × 99.1 cm.** Digital image courtesy of the Getty’s Open Content Program, The J. Paul Getty Museum, Los Angeles, CA,USA.

Although expressions such as “shed some light” or “I saw the light” figure often in our everyday speech, we do not typically contemplate light. We may marvel at its seemingly mysterious expression through auroras, rainbows, lasers, or celestial objects or find comfort in its subtler forms such as sunbeams, candles, campfires, or fireflies. Artists and scientists, by contrast, do study and manipulate light, reflection, and illumination. 

Impressionistic painters strive to capture an impression of a fleeting moment, a particular scene. Many among their numbers are considered masters for their skill at capturing light as it shimmers across surfaces. Claude Monet once described Joaquín Sorolla y Bastida Sorolla as “the painter of light above all other.”

Sorolla, was born in Valencia, Spain, in 1863. He and his infant sister Concha were orphaned when their parents died during a cholera epidemic 2 years later, and they were raised by their mother’s relatives. Early on, Sorolla seemed destined to become an artist: “When Joaquin was of an age to go to school, he manifested little inclination for his studies proper, though he revealed a stealthy and incorrigible craze for scrawling embryonic drawings in his copy books. . . .” 

In 1878, he began studies at the Fine Arts School of San Carlos, and he soon won awards at the Academy of Valencia. During 1879, the young artist traveled to Paris, where he toured exhibitions and met painters who worked in the open air, a practice that accentuated attention to light, color, and movement. That experience proved pivotal. Sorolla developed a passion for painting outdoors, preferring natural light and settings. After his military service, Sorolla subsequently attended the Fine Arts Academy in Rome on a 4-year scholarship beginning when he was 21. By the time Sorolla was 30, his paintings had been displayed across Europe and in the United States, and by the turn of the century, he was acknowledged to be among the Western world’s best living painters. 

He created many memorable paintings portraying the sun-drenched Spanish Mediterranean beaches and seascapes; he often painted portraits outside, reinvigorating the form with his fresh perspective. Sorolla’s mastery is largely accorded to his ability to depict tones and colors of sunlight, and he dedicated his life to chasing the sun as it played over the people and places of his beloved Spain. He himself acknowledged, “I hate darkness. Claude Monet once said that painting in general did not have light enough in it. I agree with him. We painters, however, can never reproduce sunlight as it really is. I can only approach the truth of it.”

Sorolla cut a dashing figure, dressed in a suit, working from a table-sized palette dabbed with an array of colors squeezed from emptied tubes, and wielding yard-long brushes. The vigor with which he worked—he often finished a painting within a few days—did not diminish even as his reputation and wealth grew: he completed more than 500 paintings during an energetic 4-year spurt.

This months’ cover image,* The Wounded Foot, *is among a series of paintings Sorolla made on the beach at Valencia. This painting focuses on 2 children, one of whom sits on the wet sand inspecting her foot, possibly injured from a jagged shell or broken shard of glass; her companion crouches, peering from under a wide-brimmed hat, perhaps offering comfort. That child’s arm falls beyond the edge of the canvas; blurred figures bob, swim, and frolic in the ocean. Reflected light scatters and glistens across the wet sand, swirling water, and foamy edges of waves. The casual composition belies the artist’s expertise in capturing transitory light and motion. The J. Paul Getty Museum notes that “The colored reflections of late afternoon light animate this beach scene and actively define the forms, from the injured child’s shoulder to the liquid sea and the figures playing in the water. The sun’s highlights on the hurt child’s hand, the sand around her foot, and her companion’s hat draw the viewer's attention to the injured limb.” 

The study of light, reflection, and illumination also seized the imagination of German scientist August Köhler, one of Sorolla’s contemporaries. In 1893, Köhler developed the microscope illumination technique that both bears his name and remains in use: Köhler illumination. This technique uniformly illuminates specimens without background glare. When this means of illumination is used on Gram-stained specimens, the defining features and structures of bacteria are readily and vividly revealed.

Köhler illumination proved ground-breaking. Advances in detection and surveillance cannot come swiftly enough as researchers and public health professionals grapple with the increasing emergence of drug resistance in bacteria. Antimicrobial drug resistance diminishes the ability to treat bacterial infections, increases risks associated with many medical procedures, and threatens animal health and agriculture. In 2014, the United States government released *The National Action Plan for Combating Antibiotic-Resistant Bacteria*, acknowledging the global scope of the problem and supporting the One Health approach to disease surveillance for pathogens of humans and animals as being critical for combating resistance to antibiotics.

Light, reflection, and illumination led an artist to capture a fleeting incident on a sunny beach in *The Wounded Foot *and inspired a scientist to find a durable solution for a problem that had vexed researchers. The growing problem of antimicrobial resistance could make treating that child’s wound today more complicated than we imagined when the widespread use of antibiotics began in the 1940s, reminding us that we must keep shedding new light on an old problem.
